# The Impact of Phenotypic and Genetic Heterogeneity on Results of Genome Wide Association Studies of Complex Diseases

**DOI:** 10.1371/journal.pone.0076295

**Published:** 2013-10-11

**Authors:** Mirko Manchia, Jeffrey Cullis, Gustavo Turecki, Guy A. Rouleau, Rudolf Uher, Martin Alda

**Affiliations:** 1 Department of Psychiatry, Dalhousie University, Halifax, Nova Scotia, Canada; 2 McGill Group for Suicide Studies, Douglas Mental Health University Institute, Montréal, Quebec, Canada; 3 Montreal Neurological Institute, Department of Neurology and Neurosurgery, McGill University, Montréal, Canada; University of Wuerzburg, Germany

## Abstract

Phenotypic misclassification (between cases) has been shown to reduce the power to detect association in genetic studies. However, it is conceivable that complex traits are heterogeneous with respect to individual genetic susceptibility and disease pathophysiology, and that the effect of heterogeneity has a larger magnitude than the effect of phenotyping errors. Although an intuitively clear concept, the effect of heterogeneity on genetic studies of common diseases has received little attention. Here we investigate the impact of phenotypic and genetic heterogeneity on the statistical power of genome wide association studies (GWAS). We first performed a study of simulated genotypic and phenotypic data. Next, we analyzed the Wellcome Trust Case-Control Consortium (WTCCC) data for diabetes mellitus (DM) type 1 (T1D) and type 2 (T2D), using varying proportions of each type of diabetes in order to examine the impact of heterogeneity on the strength and statistical significance of association previously found in the WTCCC data. In both simulated and real data, heterogeneity (presence of “non-cases”) reduced the statistical power to detect genetic association and greatly decreased the estimates of risk attributed to genetic variation. This finding was also supported by the analysis of loci validated in subsequent large-scale meta-analyses. For example, heterogeneity of 50% increases the required sample size by approximately three times. These results suggest that accurate phenotype delineation may be more important for detecting true genetic associations than increase in sample size.

## Introduction

Phenotypic misclassification reduces substantially the power to detect association, particularly in case-control studies [1]–[6]. Typically these analyses were restricted to rates of misclassification on the order of 1–5%. However, it is conceivable that complex traits may be heterogeneous with respect to genetic susceptibility and disease pathophysiology, and that the effect of phenotypic or genetic heterogeneity (henceforth referred to as “heterogeneity”) is of a larger magnitude than that of phenotypic misclassification. This is particularly relevant for psychiatric disorders [7]. The diagnosis of mental illness is based primarily on descriptive clinical criteria and is typically made in absence of laboratory diagnostic tests or other clinically relevant knowledge of individual pathophysiology. It is possible that depression, psychosis, bipolar disorder (BD), or substance abuse each represent a common phenotypic manifestation of an underlying polygenic diathesis. But it is also conceivable that these syndromes encompass diverse conditions, each with a distinct genetic basis and little overlap with the others [8]. Several such subgroups of major psychiatric disorders, including lithium responsive BD [9] and mood incongruent psychosis [10] have been proposed based on clinical, familial and biological criteria.

It is also possible that heterogeneity contributes to the discrepancy between the heritability estimates of complex diseases and the proportion of phenotypic variance explained by the identified loci in genome-wide association studies (GWAS). Indeed, the estimated effect sizes of genetic associations in GWAS of complex traits are significantly reduced by imprecise phenotyping [11], [12]. This discrepancy appears to be of larger magnitude in psychiatric disorders than in other complex traits [13]. For instance, the heritability of BD has been estimated to be as high as 85% [14], [15]. But only a smaller fraction of BD heritability is accounted for by loci identified through GWAS, even when considering all GWAS polymorphisms simultaneously [16]–[19].

Little attention has been given so far to the extent of the effect of heterogeneity on genetic association findings in common complex diseases. Testing the impact of heterogeneity on GWAS findings requires extensive knowledge of the pathophysiology and genetic architecture of the common trait under study. This assumption makes psychiatric disorders unsuitable for such analysis. On the other hand, another common disease such as diabetes mellitus (DM) is suitable for testing the impact of heterogeneity. The two types of DM were distinguished only in the late 1930s [20], each characterized by distinct pathophysiology [21] and genetic architecture. If a diagnosis of DM was based on high blood glucose alone, DM type 1 (T1D) cases could not be differentiated from DM type 2 (T2D) cases. The state of DM classification prior to 1930 may well approximate the state of knowledge about psychiatric disorders in the beginning of the 21^st^ century.

Here we investigate the impact of heterogeneity on the statistical power of GWAS. To do so, we first performed a computer simulation study. Next, we analyzed the Wellcome Trust Case-Control Consortium (WTCCC) data for DM T1D and T2D. We combined varying proportions of individual data from each of the diabetes subtypes to examine the impact of heterogeneity on the strength and statistical significance of association and compared these results with those previously found in the WTCCC study. Heterogeneity reduced the statistical power to detect genetic association and greatly decreased the estimates of risk attributed to genetic variation.

## Materials and Methods

### Simulation of Case-Control Association Analysis Under Heterogeneity

To study the impact of heterogeneity on GWAS findings, we simulated case-control data assuming increasing (from 10% to 90% in 10% steps) phenotypic admixture. Admixture (indicated as β) was the proportion of “non-cases” (i.e. controls) in the case group. Specific genetic models of disease prevalence were used in order to simulate genotypes for case and control populations. We used two basic single-locus genetic models: dominant [22] and multiplicative (or log additive) [23]. The model parameters were the following: population prevalence for the disorder (0.001, 0.01, 0.05, 0.1), minor allele frequency (MAF) (0.01, 0.05, 0.1, 0.2, 0.3, 0.4, 0.5), and the relative risk of the disorder for the minor allele (1.1, 1.2, 1.3, 1.5, 2, 5, 10). We used a Monte Carlo procedure to simulate cases and controls under all combinations of the parameters listed above. Genotypes were assigned using one of two probability distributions, according to the group (case or control) that each individual came from. Using a script in R software (version 2.13.2), we performed two sets of simulations. First, we determined the minimum sample size needed to detect an association in 90% of trials at a significance level set at p<5×10^−8^. The sample size obtained at each iteration of the simulation was specified according to a binary search that terminated once 90% power (over 10,000 replicates) was achieved, or when the sample size limit of 1,000,000 was reached. In the second set of simulations, we studied the impact of β on the statistical power to detect association at p<5×10^−8^. To this end we generated 10,000 cases and controls, replicated 1,000 times, for each combination of parameters. For each genetic model (dominant and multiplicative) we obtained case-control frequency tables. Using χ^2^ and the Cochran-Armitage-Trend-Test (CATT) module implemented in R we calculated p-values and odds ratios (ORs) for increasing levels of β.

### Analysis of WTCCC Type 1 and Type 2 Diabetes Data Under Heterogeneity

We accessed and downloaded all of the genotypic data available for the two control populations (the 1958 Birth Cohort (58C) and the UK National Study (NBS)) and for the T1D and T2D cohorts from the WTCCC website.

A number of quality control (QC) steps were performed on this data in the original WTCCC GWAS study [24]. Individuals and SNPs that were retained had passed each of the following exclusion criteria: 1) missing data rate>3% per sample across all SNPs; 2) heterozygosity>30% or <23%; 3) discrepancies in ID information; 4) ancestry control (outliers after multi-dimensional scaling); 5) duplicated samples (identity>99%); 6) relatedness (86%–96% identity); 7) missing data rate>5% per SNP; 8) missing data rate>1% when MAF<5%; 9) Hardy-Weinberg exact p-value<5.7×10^−7^ (in 2,938 controls) [24].

Since the QC analysis in the original study was performed across ∼14,000 subjects (compared to the ∼7,000 available for the present study), we also applied exclusion lists supplied in the WTCCC data in addition to in-house QC. The scripts used for QC are available online.

Two recoded phenotype files were created each containing 2,938 control individuals (the combination of all the filtered 58C and NBS individuals) and the 1,963 T1D or the 1,924 T2D cases, respectively. Next, we performed the WTCCC standard case-control association analysis (χ^2^) to confirm the validity of the QC steps using PLINK [25]. Results of this analysis are outlined in Table 1.

To investigate the effect of heterogeneity, we examined the 20 SNPs most significantly associated with T1D or T2D, respectively. These SNPs are independent and in linkage equilibrium to each other, with the exception of rs9939609 and rs7193144 within the FTO gene which are in strong linkage disequilibrium (R^2^ = 0.97, D’ = 1) and are both associated with T2D. Further, we studied 21 and 16 polymorphisms found associated in subsequent large-scale meta-analyses for T1D [26]–[29] and T2D [30], respectively. The selected SNPs were genotyped in the WTCCC study and were required to have p<10^−6^ in at least one meta-analysis study. We created alternate phenotype files in order to simulate heterogeneity. Let N_1_ and N_2_ denote the respective totals of T1D and T2D cases. At each 10% increment of β, we removed N_1_*β subjects randomly selected from the T1D population and replaced them with N_1_*β subjects randomly chosen from the T2D population. Similarly, we analyzed T2D data, replacing N_2_*β T2D with T1D cases. For each of T1D and T2D, 100 alternate samples were created at each 10% increment of β. Association analyses were then run using the χ^2^ test in PLINK [25] on each alternate phenotype file. We took the −log (p-value) from each of the 100 association analyses at each β level, and then calculated the arithmetic mean of the −log (p-value). The scripts developed for the analyses presented in the study are available at the following URL: http://web.cs.dal.ca/~cullis/heterogeneity/.

## Results

### Simulated Case-Control Association Analysis Under Heterogeneity

**Figure 1:**
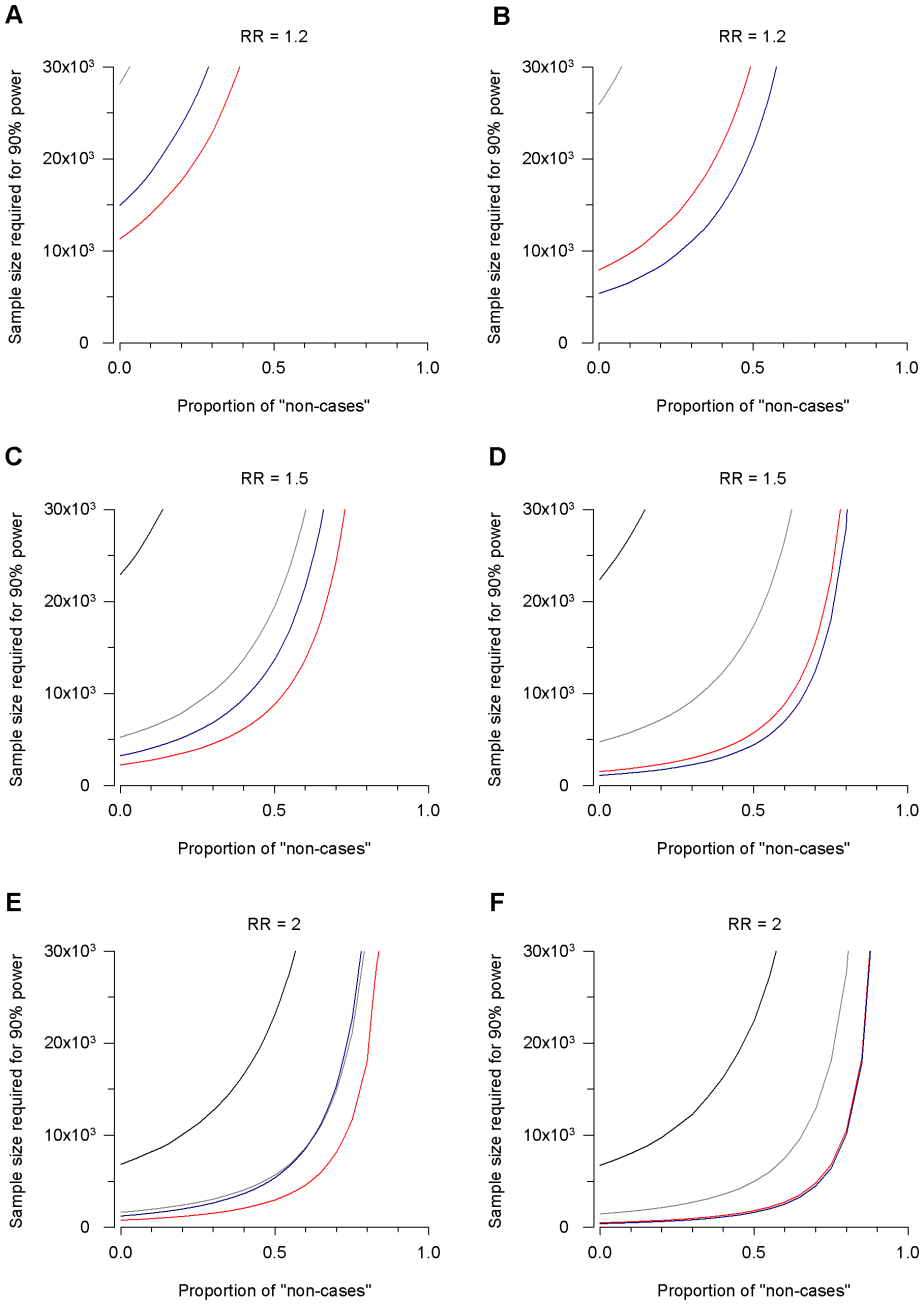
The impact of heterogeneity on the sample size (cases and controls) required for 90% of statistical power. The minimum sample size to achieve to detect association was calculated in simulated case-control data with increasing proportion of “non-cases” considering a disease prevalence of 0.01. Data are reported for minor allele frequencies (MAF) of 0.01 (black), 0.05 (grey), 0.2 (red) and 0.5 (blue). The results are reported for dominant (panels A, C, and E) and multiplicative (panels B, D, and F) genetic models. RR = relative risk.

We simulated case-control data assuming increasing β. We present the results for a disease prevalence of 0.01. This value is relevant to complex traits such as BD or schizophrenia. Results for other prevalence values can be found in the Table S1 and S2. In all analyses, the admixture level significantly affected the sample size required to reach 90% power at the significance level of p<5×10^−8^. Specifically, there was a substantial loss of statistical power that was disproportionately larger than the degree of heterogeneity. An increase in the proportion of “non-cases” resulted in a non-linear increase of the sample size needed to achieve 90% of statistical power. As shown in Figure 1, this effect was evident both in the dominant and multiplicative models and for different relative risks (RR = 1.2, 1.5 and 2). For instance, with β at 50%, the sample size needed to achieve the same statistical power without admixture was three times larger, particularly with genetic effect sizes equal to or larger than 1.5.

**Figure 2:**
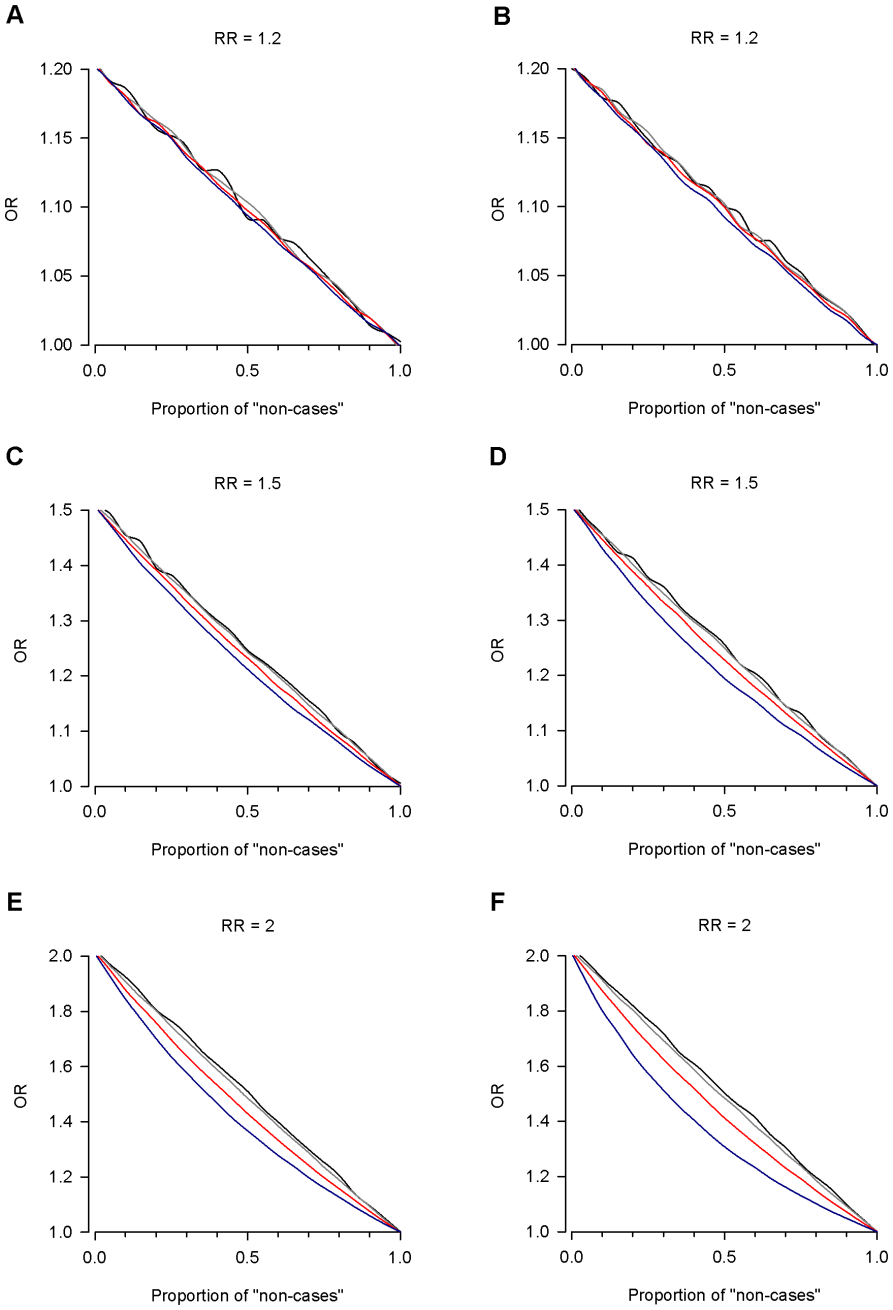
The impact of heterogeneity on the estimation of the genetic effect size. Odds ratios from simulated case-control data were calculated for each step of admixture. Data are reported for minor allele frequencies (MAF) of 0.01 (black), 0.05 (grey), 0.2 (red) and 0.5 (blue). The results are reported for dominant (panels A, C, and E) and multiplicative (panels B, D, and F) genetic models. RR = relative risk; OR = odds ratio.

Heterogeneity also resulted in a marked reduction of the estimated effect size which is reflected here as a decrease in the estimated ORs. This reduction in effect size was found to be non-linear in relation to the degree of β and was more pronounced for larger relative risk values (Figure 2).

### Application to WTCCC Type 1 and Type 2 Diabetes Data

**Figure 3:**
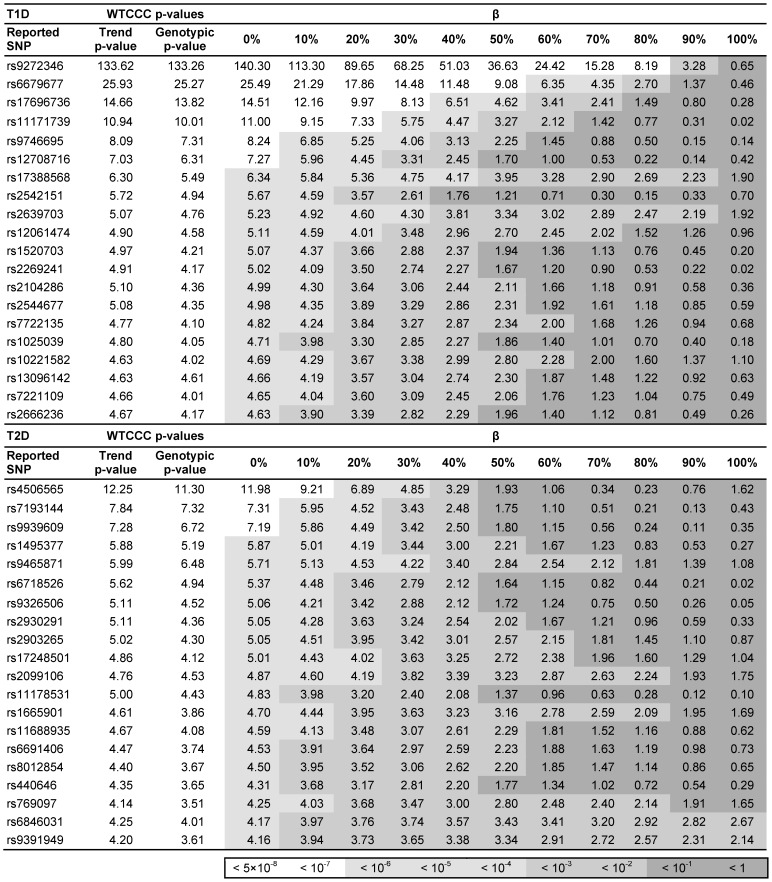
Genome-wide analysis of the Wellcome Trust Case-Control Consortium (WTCCC) data for diabetes type 1 (T1D) and type 2 (T2D) under heterogeneity: twenty most significant associations [−log(p-values)]. SNP = single nucleotide polymorphism; β = admixture.

To further test the validity of the results identified in the computer simulation we analyzed the WTCCC T1D and T2D data [24]. In both datasets we examined the 20 most significantly associated SNPs. Of these, 7 loci for T1D and 3 for T2D reached genome-wide significance and an additional 13 and 17 loci, respectively, showed moderate association. The results of this analysis are outlined in Figure 3. The strength of the association for T1D decreased substantially as the proportion of T2D cases in the sample was increased. Most of the significantly associated SNPs became equivocal at a relatively low degree of β (between 20% and 30%). Only the association of *HLA-DRB1* with T1D [31] was robust to the effects of heterogeneity, with a significant effect present with up to 90% of the sample made up of T2D cases. Similarly, the strength of the association signals for T2D progressively diminished as T1D cases gradually replaced the “true” cases of T2D. Further support came from the analysis of loci found associated in subsequent meta-analyses of T1D and T2D (Figures S1 and S2). Again, with increasing degree of β the magnitude of association of the SNPs declined substantially.

## Discussion

The purpose of this study was to quantify the impact of heterogeneity in the analysis and interpretation of GWAS findings. We showed that the presence of heterogeneity (presence of “non-cases”) reduced both the statistical power as well as the observed risks attributed to susceptibility alleles or genotypes. These findings were supported by the analysis of both simulated case-control and GWAS data from WTCCC T1D and T2D cohorts [24]. We also tested our hypothesis by analyzing loci replicated and validated in large-scale meta-analyses irrespective of their association strength in the WTCCC study, as these are more likely to represent true associations. The same pattern was observed for these loci: the magnitude of association declined substantially with increasing heterogeneity.

Several findings deserve comment. First, irrespective of the various parameters and genetic models tested, the absence of heterogeneity in samples allows for a power to detect association that would require a far larger sample size if a medium or high level of heterogeneity were present. A recent study examining the impact of diagnostic misclassification in genetic studies by Wray et al. [32] has reached similar results with a different method. In the Supplementary Information to their paper, Wray et al. [32] provide an analytical solution that allows the calculation of statistical power under misclassification without computer simulation. These observations are consistent with the findings of pharmacogenomic GWAS. Indeed, the robustness of the phenotypic measure of treatment response given by specific biomarkers (for example identifiable in serum) allowed the identification of significant association signals even with relatively small sample sizes [33]. In addition, pharmacogenetic traits have not been subject to selection and so larger effect sizes may exist compared to complex traits.

In the absence of more reliable measures (for instance diagnostic tests) in more heterogeneous complex traits – including many psychiatric and neurological diseases – researchers have obtained promising results by selecting more homogeneous subgroups of patients such as responders to lithium treatment in BD [34], or early illness onset in Alzheimer’s disease [35]. This strategy can significantly decrease the number of cases to collect for adequately powered genetic studies.

We have also shown that the presence of heterogeneity strongly influences the estimates of the effect sizes. This result suggests that the effect sizes of marginally significant variants identified in GWAS might be larger if they were instead examined in more homogeneous samples. This is in agreement with previous studies pointing to the importance of accurate and stringent phenotypic definition in GWAS data [11], [12]. Interestingly, van der Sluis et al. [12] employed simulation studies to show that accurate modelling of phenotypic information improved the estimation of the genetic variants effect size. Further, Evangelou et al. [11] found empirical support for importance of phenotypic definition in the analysis of GWAS data in HIV-1 infected subjects. They showed that the observed genetic effects in HIV-1 seroconverters (a more stringent phenotype) were larger than in seroprevalent subjects. Using simulation and real data both studies consistently showed how accurate phenotyping increased the power to detect association signals and to estimate correctly their effect size.

Here we observed that as the degree of heterogeneity increased, so did the minimum sample size required to achieve sufficient statistical power. This effect was particularly evident for genotypic relative risk values on the order of 1.2 and 1.5 and for common variants (MAF>0.05). Interestingly, for a complex trait such as BD, most of the reported genetic risk variants have an associated effect size of approximately 1.5 or below [24], [36]–[42]. At least in clinically heterogeneous disorders, it is conceivable that even collecting large sample sizes could only partially compensate for the loss of statistical power in GWAS. In this context it appears crucial to focus on the steps preceding the GWAS. Careful clinical history from all available sources, consensus diagnosis, validity of the phenotypic measures used, evaluation of the inter-rater agreement and reliability and use of prospective design could all help in overcoming the issue of phenotypic heterogeneity.

In conclusion, we demonstrated that heterogeneity can have a major impact on GWAS findings. The extent of its effect is of large magnitude and, unexpectedly, affects significantly even loci robustly associated with the trait under study. We showed that even a relatively low proportion of “non cases” (20%) can dilute the observed genetic effect size for common variants (with MAF>0.05) targeted in the current GWAS analyses. The partial failure of GWAS to detect a substantial proportion of the heritability of genetic complex diseases could be a consequence of the presence of a high degree of heterogeneity.

## Supporting Information

Figure S1 Impact of the admixture of diabetes type 1 (T1D) and type 2 (T2D) on Wellcome Trust Case-Control Consortium (WTCCC) T1D findings confirmed in large scale meta-analyses [−log(p-values)]. SNP = single nucleotide polymorphism; β = admixture; nr = not reported.
(TIF)

Figure S2 Impact of the admixture of diabetes type 1 (T1D) and type 2 (T2D) on Wellcome Trust Case-Control Consortium (WTCCC) T2D findings confirmed in large scale meta-analysis [−log(p-values)]. SNP = single nucleotide polymorphism; β = admixture; nr = not reported. *p-value reported in the Zeggini et al. [1] study. Reference: 1. Zeggini E, Weedon MN, Lindgren CM, Frayling TM, Elliott KS, et al. (2007) Replication of genome-wide association signals in UK samples reveals risk loci for type 2 diabetes. Science 316: 1336–1341.
(TIF)

Table S1 Results of case-control simulation to determine the minimum sample size needed to detect an association in 90% of trials at a significance level set at p<5×10^−8^ with increasing levels of admixture (β). Results are presented for dominant and multiplicative genetic models with the following parameters: population prevalence for the disorder (0.001, 0.01, 0.05, 0.1), minor allele frequency (MAF) (0.01, 0.05, 0.1, 0.2, 0.3, 0.4, 0.5), and the relative risk of the disorder for the minor allele (1.1, 1.2, 1.3, 1.5, 2, 5, 10).
(XLS)

Table S2 Case-control simulation results of the impact of increasing level of admixture (β) on the statistical power to detect association at p<5×10^−8^. Results are presented for dominant and multiplicative genetic models with the following parameters: population prevalence for the disorder (0.001, 0.01, 0.05, 0.1), minor allele frequency (MAF) (0.01, 0.05, 0.1, 0.2, 0.3, 0.4, 0.5), and the relative risk of the disorder for the minor allele (1.1, 1.2, 1.3, 1.5, 2, 5, 10).
(XLS)
