# Supplementary figures and images for: The Impact of Phenotypic and Genetic Heterogeneity on Results of Genome Wide Association Studies of Complex Diseases
Source: PLoS One. 2013 Oct 11;8(10):e76295. doi: 10.1371/journal.pone.0076295 (PMC3795757; doi:10.1371/journal.pone.0076295)

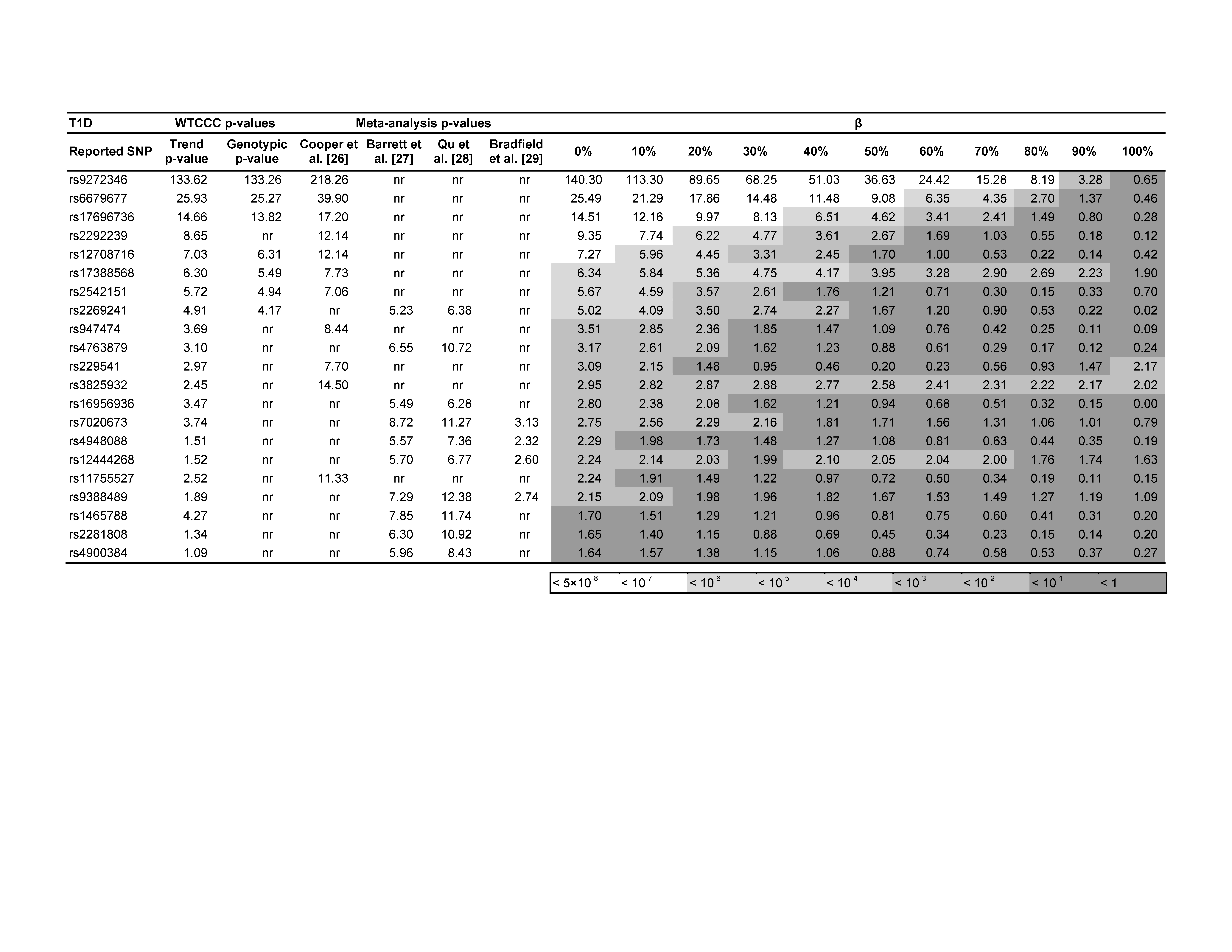

Supplement: Figure S1 — Impact of the admixture of diabetes type 1 (T1D) and type 2 (T2D) on Wellcome Trust Case-Control Consortium (WTCCC) T1D findings confirmed in large scale meta-analyses [−log(p-values)]. SNP = single nucleotide polymorphism; β = admixture; nr = not reported. (TIF) [file pone.0076295.s001.tif]

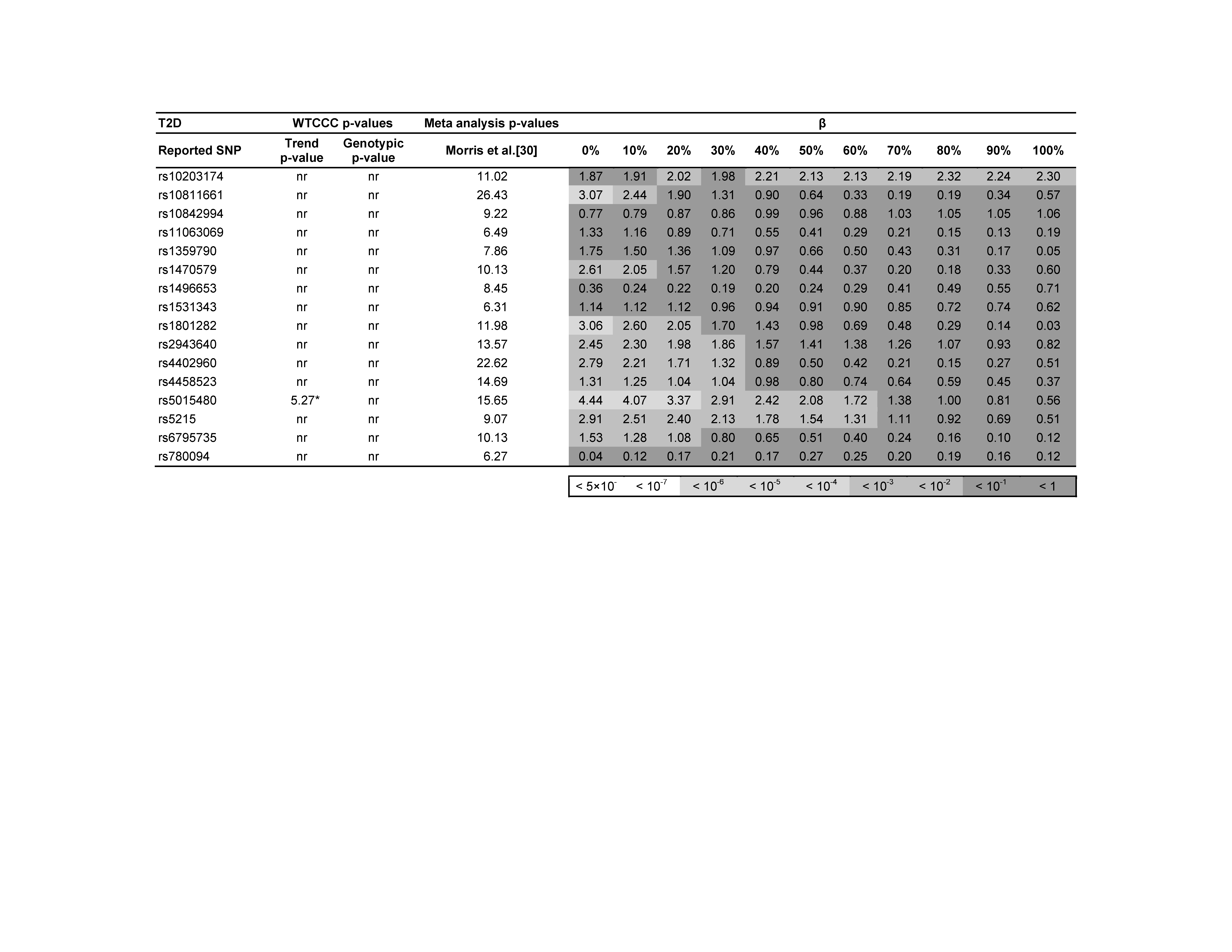

Supplement: Figure S2 — Impact of the admixture of diabetes type 1 (T1D) and type 2 (T2D) on Wellcome Trust Case-Control Consortium (WTCCC) T2D findings confirmed in large scale meta-analysis [−log(p-values)]. SNP = single nucleotide polymorphism; β = admixture; nr = not reported. *p-value reported in the Zeggini et al. [1] study. Reference: 1. Zeggini E, Weedon MN, Lindgren CM, Frayling TM, Elliott KS, et al. (2007) Replication of genome-wide association signals in UK samples reveals risk loci for type 2 diabetes. Science 316: 1336–1341. (TIF) [file pone.0076295.s002.tif]
